# A model for predicting bacteremia species based on host immune response

**DOI:** 10.3389/fcimb.2025.1451293

**Published:** 2025-02-18

**Authors:** Peter Simons, Virginie Bondu, Laura Shevy, Stephen Young, Angela Wandinger-Ness, Cristian G. Bologa, Tione Buranda

**Affiliations:** ^1^ Department of Pathology, University of New Mexico Health Sciences Center, Albuquerque, NM, United States; ^2^ Division of Infectious Diseases, Department of Internal Medicine, University of New Mexico, Health Sciences Center, Albuquerque, NM, United States; ^3^ Tricore Research Laboratories, Albuquerque, NM, United States; ^4^ Translational Informatics Division, Department of Internal Medicine, University of New Mexico Health Sciences Center, Albuquerque, NM, United States

**Keywords:** GTPases, Rac1 activation, bacteremia diagnosis, PLS-DA algorithm, G-Trap assay, flow cytometry, sepsis

## Abstract

**Introduction:**

Clinicians encounter significant challenges in quickly and accurately identifying the bacterial species responsible for patient bacteremia and in selecting appropriate antibiotics for timely treatment. This study introduces a novel approach that combines immune response data from routine blood counts with assessments of immune cell activation, specifically through quantitative measurements of Rho family GTPase activity. The combined data were used to develop a machine-learning model capable of distinguishing specific classes of bacteria and their associations.

**Methods:**

We aimed to determine whether different classes of bacteria elicit distinct patterns of host immune responses, as indicated by quantitative differences in leukocyte populations from routine complete blood counts with differential. Concurrently, we conducted quantitative measurements of activated Rac1 (Rac1•GTP) levels using a novel ‘G-Trap assay’ we developed. With the G-Trap, we measured Rac1•GTP in peripheral blood monocytes (PBMC) and polymorphonuclear (PMN) cells from blood samples collected from 28 culture-positive patients and over 80 non-infected patients used as controls.

**Results:**

Our findings indicated that 18 of the 28 patients with bacteremia showed an increase of ≥ 3-fold in Rac1•GTP levels compared to the controls. The remaining ten patients with bacteremia exhibited either neutrophilia or pancytopenia and displayed normal to below-normal Rac1 GTPase activity, which is consistent with bacteria-induced immunosuppression. To analyze the data, we employed partial least squares discriminant analysis (PLS-DA), a supervised method that optimizes group separation and aids in building a novel machine-learning model for pathogen identification.

**Discussion:**

The results demonstrated that PLS-DA effectively differentiates between specific pathogen groups, and external validation confirmed the predictive model's utility. Given that bacterial culture confirmation may take several days, our study underscores the potential of combining routine assays with a machine-learning model as a valuable clinical decision-support tool. This approach could enable prompt and accurate treatment on the same day that patients present to the clinic.

## Introduction

Timely identification of species causing bacteremia in different patients is challenging for healthcare providers and delays effective treatment. Blood culture is commonly used to identify bacterial species associated with an infection but has limitations. Blood culture is only 30% accurate due to several factors, including blood volume restrictions, timing of blood draw, antibiotic treatment, and viable organisms. False positives can also occur due to contamination, particularly with *Staphylococcus epidermidis* (*S. epidermidis)*, part of the normal skin microbiota (Pancholi et al., 2018; Sweeney et al., 2019; Tamma et al., 2019; Ducharme et al., 2020; Ehren et al., 2020; He et al., 2021); Park et al. (2021). Most infections may not appear in the bloodstream, and specific tests take time to produce accurate results. Blood cultures are prone to insensitivity and delayed results that take days. Therefore, an unmet need remains for accurately and rapidly identifying the bacterial species that cause patient bacteremia to enable appropriate and timely antibiotic administration.

The immune system of vertebrates protects against invading pathogens through leukocyte activation. Hence, strategies targeting the host response have improved sepsis diagnosis and treatment (de Jong et al., 2016; Huang et al., 2018; Iankova et al., 2018; Gunsolus et al., 2019). Studies have found that combining mRNA panels monitoring host immune responses with machine learning can accurately identify the type and location of an infection (Gunsolus et al., 2019; Sweeney et al., 2019; Ducharme et al., 2020; He et al., 2021). However, mRNA panels cannot differentiate between bacterial species. A complete blood count with differential (CBC with differential) is a standard blood test that evaluates the levels of red and white blood cells, hemoglobin, hematocrit, platelets, and cell size, shape, and color. It is an affordable and expected standard of clinical care that provides information on immune activation status.

Innate immunity recognizes pathogenic microorganisms and helps activate the secretion of secondary signals that stimulate adaptive immunity. Toll-like receptors (TLRs) are essential membrane-spanning recognition receptors on the surface of immune cells that identify pathogenic biomarkers such as bacterial lipopolysaccharides or diacylated lipoproteins expressed on the outer membrane of gram-negative and gram-positive bacteria, respectively (Koch and Zacharowski, 2009; Reynolds et al., 2012). The encounter between bacteria and TLR-induced host responses activates transcription factors, including nuclear factor-kappa B, p38 mitogen-activated protein kinase, and interferon regulatory factors, crucial for initiating proinflammatory signaling pathways (Kawai and Akira, 2007). Depending on the assessment of host-pathogen risk factors, proinflammatory cytokines such as tumor necrosis factor, interleukin-1, and interleukin-6 are secreted to stimulate the production and recruitment of neutrophils and macrophages. Thus, changes in immune cell counts have long provided an important clinical aid in the diagnosis of bacterial infection.

**Figure 1:**
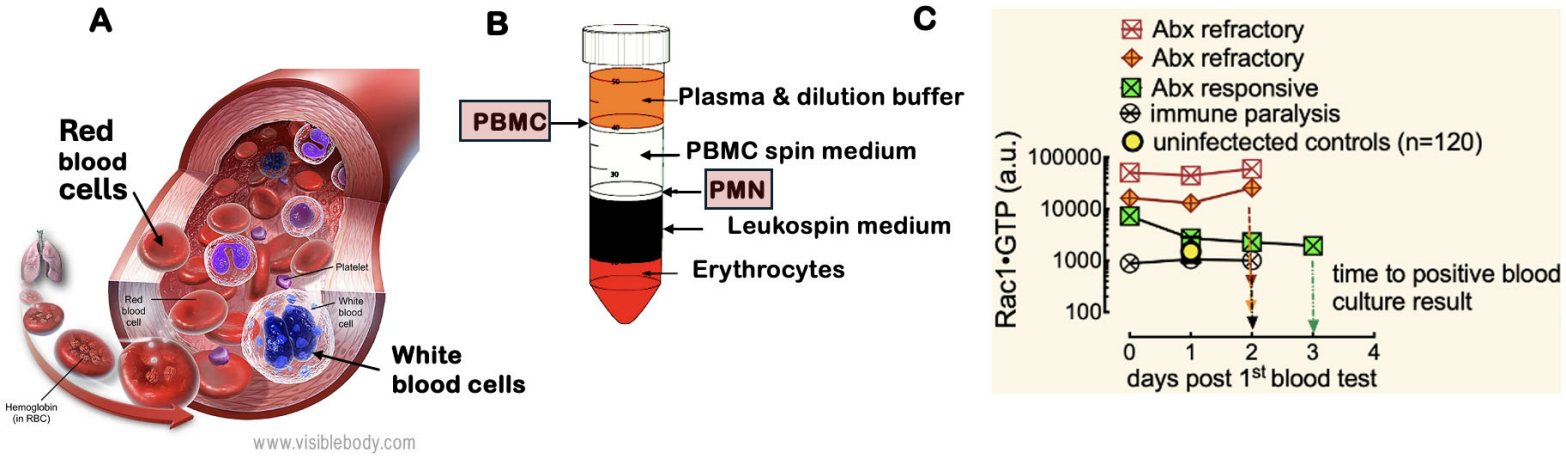
White blood cell motility is critical for the inflammatory response to infection. The regulation of the actin cytoskeleton by Rho family GTPases (e.g., Rac1) and their kinases is crucial for leukocyte migration to sites of inflammation, making them essential biomarkers for identifying sources of bacteremia. **(A)** White blood cells include PMN: polymorphonuclear cells (neutrophils, eosinophils, and basophils) and PBMC: peripheral blood mononuclear cells (lymphocytes, monocytes, natural killer cells, and dendritic cells). Image courtesy of Visible Body (https://www.visiblebody.com/learn/biology/blood-cells/blood-overview). A complete blood count with differential reflects changes in leukocyte counts caused by various pathogens. **(B)** The separation of whole blood components using a Ficoll gradient allows for the isolation of PMNs and PBMCs, which are subsequently analyzed with the G-Trap assay that quantitatively measures activated Rac1 (Rac1•GTP) levels in lysates of PBMCs and PMNs. **(C)** Serial analysis of PBMCs from representative patients with an active immune response to bacteremia revealed that two patients were resistant to broad-spectrum antibiotic treatment, while one responded positively. Additionally, one patient exhibited immune suppression, as indicated by low Rac1•GTP levels.

Ras-related C3 botulinum toxin substrate 1 (Rac1) is a key member of the Rho family of small guanosine triphosphatases (GTPases) and is crucial for host immunity. Rac1 is essential in actin cytoskeleton reorganization, superoxide production, and cell migration, central to a productive host immune response (Bokoch, 2005). Rac1 mobilizes leukocytes through the nuclear factor-kappa B pathway and promotes the production of pro-inflammatory cytokines, chemokines, and adhesion molecules. By integrating immune receptor signals, activated Rac1 regulates the directed movement and adhesion of immune cells, which is vital for blood leukocyte chemotaxis and tissue extravasation during infections (**Figures 1A–C**) (Benvenuti et al., 2004; Tybulewicz and Henderson, 2009; Ridley, 2011). Rac1 GTPase responds to chemotactic factors and cytokine receptor activation by transitioning from an inactive guanosine diphosphate-bound state to an active guanosine triphosphate (GTP)-bound state. This activation process is facilitated by guanine nucleotide exchange factors and GTPase-activating proteins, making the activation status of Rac1 a sensitive indicator of immune cell activation (Dharmawardhane and Bokoch, 1997; Perona et al., 1997; Van Aelst and D'Souza-Schorey, 1997; Jones et al., 1998; Scheffzek et al., 1998; DerMardirossian and Bokoch, 2005; Oakley et al., 2009; Tong and Tergaonkar, 2014). Specifically, GTP-bound Rac1 (Rac1•GTP) interacts with effector proteins such as p21-activated kinase (PAK1). The binding of Rac1•GTP to PAK1 activates its kinase activity, which is necessary for immune cell motility, structural changes, and cytoskeletal alterations (Henning and Cantrell, 1998; Cantrell, 2003; Bokoch, 2005; Dumont et al., 2009; Cherfils and Zeghouf, 2013; Lemichez and Aktories, 2013; Saoudi et al., 2014; Tong and Tergaonkar, 2014). Therefore, PAK1 binding can be used to monitor Rac1 activation status.

In our study, we utilized a novel, highly quantitative G-Trap assay we developed that is based on PAK1-functionalized beads (Simons et al., 2018) to measure Rac1•GTP levels in polymorphonuclear leukocytes (PMNs) and peripheral blood mononuclear cells (PBMCs) (**Figure 1D**). Blood samples were from patients with culture-confirmed bacteremia and control patients with non-infectious disease. Our research included samples from patients infected with various bacterial species known to cause bacteremia. The bacteria included gram-positive organisms, such as *Staphylococcus aureus (S. aureus), Methicillin-resistant Staphylococcus aureus (*MRSA*), Streptococcus pyogenes (S. pyogenes), Streptococcus pneumoniae (S. pneumoniae)*, and *Staphylococcus lugdunensis (S. lugdunensis*). The gram-negative bacteria included *Escherichia coli (E. coli), Enterobacter aerogenes (E. aerogenes), Klebsiella pneumoniae (K. pneumoniae), Pasteurella multocida (P. multocida), and Pseudomonas aeruginosa (P. aeruginosa).* Our study was based on the premise that host immune responses to bacterial infections present in distinct patterns dependent on the bacterial species. We analyzed the host immune response patterns using a weighted linear combination of immune variables found in peripheral blood. Specifically, we focused on neutrophil, lymphocyte, and monocyte counts, along with Rac1•GTP levels in PMNs and PBMCs. Together, these factors formed a minimum basis set for our analyses.

Using the immune responsiveness data for Partial Least Squares Discriminant Analysis (PLS-DA) (Eriksson et al., 1999), we can rapidly identify and discriminate differences between the pathogens responsible for bacteremia in a collection of peripheral blood samples from a heterogeneous group of patients. We evaluated the efficacy of identifying unknown bacteremia species using a machine-learning training data set (Lien et al., 2022) and test samples. Our model classified all test samples accurately, demonstrating the potential for *rendering clinical decision support* in the timely and precise administration of antimicrobial therapy and limiting the spread of antibiotic resistance.

## Results

### Baseline measurements of control samples: Rac1•GTP levels in uninfected patient PBMCs are sensitive to environmental allergies, while levels in PMNs remain stable

**Figure 2:**
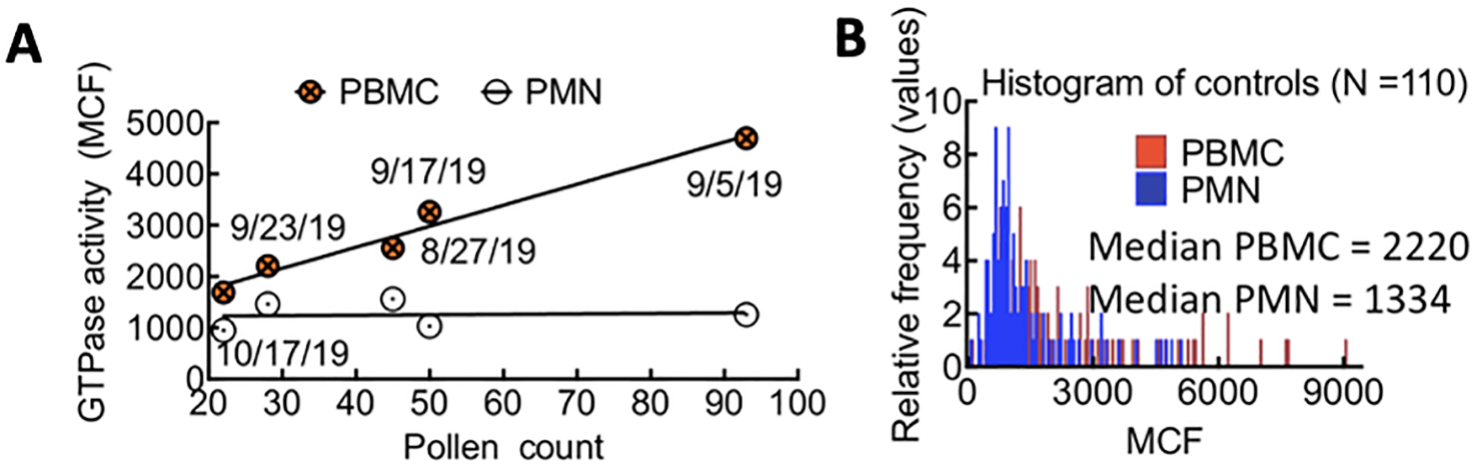
**(A)** Correlated changes in GTPase activity in PBMCs and pollen count on the day of blood sample collection. Each day represents the average of 12 patient samples. Pearson correlation (ρ = 0.78, p = 0.05). **(B)** Overlapping histograms of Rac-1 GTPase activity in leukocytes from TriCore diagnostic blood draws of uninfected patients and measured using the G-Trap assay. The fluorescence value for each patient is represented as the Median Chanel Fluorescence (MCF) readout from the G-Trap assay. The median values for polymorphonuclear neutrophils (PMN) and peripheral blood mononuclear cells (PBMC) in the patient population tested are 1334 and 2220, respectively.

We examined peripheral blood samples from over 80 non-infected individuals to establish a baseline for GTPase activity in control subjects. Blood samples were collected from August to December, with approximately 12 distinct patient samples analyzed weekly. We observed that the median GTPase activity in PBMCs (i.e., lymphocytes, monocytes, natural killer cells (NK cells), or dendritic cells) fluctuated weekly in August-October. In contrast, the GTPase activity of PMNs (i.e., neutrophils, basophils, and eosinophils) remained stable (**Figures 2A, B**). We hypothesized that changes in local pollen counts influenced innate lymphoid cell (ILC)-mediated allergic responses, causing the observed variations in Rac1•GTP in PBMCs. (Arango Duque and Descoteaux, 2014; Panda and Colonna, 2019) In support of our hypothesis, a plot of pollen counts (Weather.com) in Albuquerque, New Mexico, on the day blood samples were collected demonstrated a robust and significant Pearson correlation (ρ = 0.78, p = 0.005) between median GTPase activity in PBMC samples and pollen counts. We used this signal variability to establish a baseline signal range for uninfected patients in Albuquerque, NM.

### Bacteria classification and measurement of influence on host immune responses to infection

Gram-positive and gram-negative bacteria elicit immune responses that manifest as changes in white blood cell counts and are linked to changes in Rac1•GTP levels in leukocytes, triggering their activation and mobilization to sites of infection. It was our goal to test if data obtained from routine blood counts combined with assessments of immune cell activation, specifically through quantitative measurements of Rho family GTPase activity, could differentiate responses to individual bacterial species.

**Figure 3:**
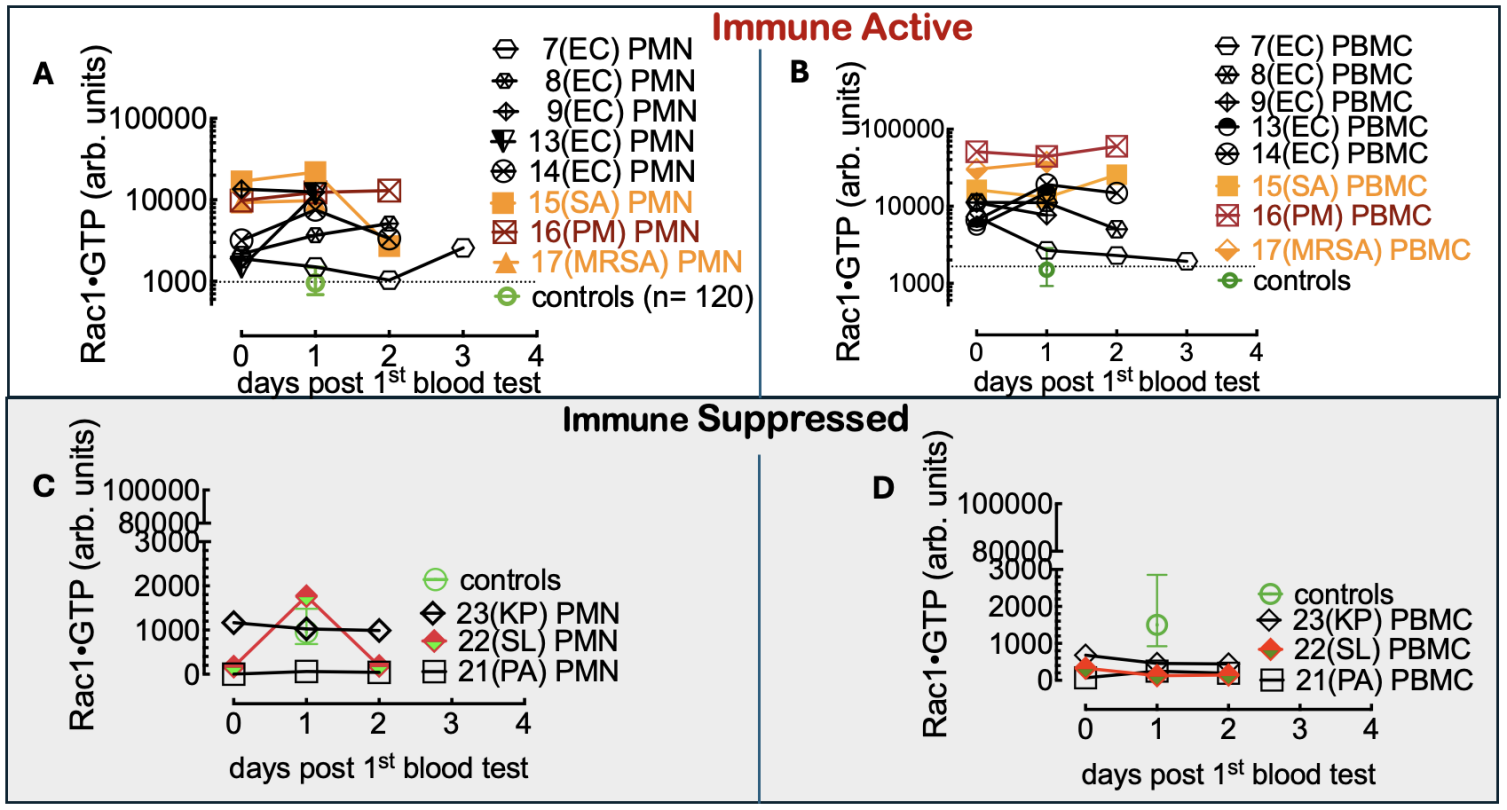
**(A, B)** Rac1•GTP content measured in PBMCs and PMNs from patients with bacteremia reveals GTPase activities ≥ 3-fold above controls. Rac1•GTP was measured in lysates from serial peripheral-blood samples (PMN or PBMC as indicated on the plots) recovered from Tricore Research laboratories post-admission and before a positive culture result. Each patient is identified numerically. Infecting pathogen abbreviations in parentheses on the plot are identified as follows: *P. multocida (*PM*)* and *E*. *coli* (EC) are gram-negative. Gram-positive species are *S. aureus* (SA) and *methicillin-resistant S. aureus* (MRSA).**(C, D)** Representative graphs of Rac1•GTP levels in serial lysates from PBMCs or PMNs from patients. Rac1•GTP levels were < 3-fold above and often below control levels due to host immune suppression. Gram-negative species: *P. aeruginosa* (PA), *K*. *pneumoniae* (PA). Gram-positive species *S. lugdunensis* (SL). Datapoints on each plot represent the standard error of the mean.

**Figure 4:**
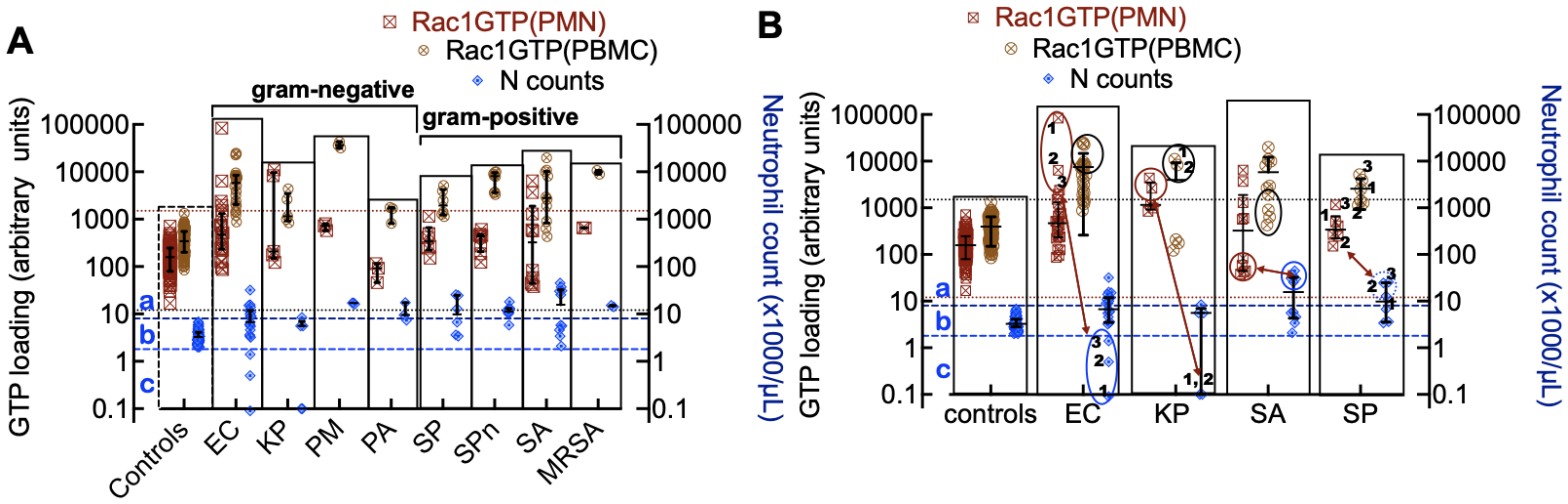
**(A)** Rac1•GTP levels measured in individual patient blood leukocytes (PBMC and PMN) who were diagnosed with bacterial infections due to gram-negative strains (*E. coli* (EC, n=22), *K*. *pneumoniae* (KP, n=7), *P. multocida* (PM, n=3) *P. aeruginosa* (PA, n=3)) or gram-positive strains (*S. pyogenes* (SP, n=12), *S. pneumoniae* (SPn, n=8), *S. aureus (*SA, n=10), MRSA (n=2)). **(B)** Depleted cell counts in late-stage samples are caused by cell-ingested EC (1, 2, 3) and KP (1, 2), which kills the cells. To suppress an overactive immune system, SA and SP can inactivate neutrophil function, which may be manifested as notably elevated neutrophil counts (neutrophilia). The double-headed arrows match the low neutrophil counts with Rac1•GTP levels in gram-negative bacteria. Gram-positive bacteria correlate between peak numbers of neutrophils and Rac1•GTP suppression. The error bars represent the median with the interquartile range. **(A, B)** The two top horizontal dotted lines denote the upper limit of GTPase activity found in control PBMCs and the lower limit of GTPase activity found in PMNs. The two bottom dashed lines represent the upper and lower limits of control neutrophil counts. **(a)** The zone containing neutrophils is considered to be activated or deactivated based on GTPase activity and elevated cell counts. **(b)** Zone of impaired immune cell mobilization. **(c)** Zone of phagocytosis-induced cell death (Kobayashi et al., 2018). Ellipsoids in panel B indicate depleted or elevated neutrophil counts and associated GTPase response.

To do so, we first compared Rac1•GTP levels in PMNs and PBMCs from the uninfected patients (81 included in the final analysis) to those found in 61 serial samples drawn from 28 patients with blood culture-positive bacterial infections. Samples were from patients with bacteremia caused by 10 different gram-positive or gram-negative bacterial species. The range of Rac1•GTP-mediated immune responses to infection was determined by plotting the Rac1•GTP levels in cell populations of activated PBMCs and PMNs from representative serial patient samples and found to be ≥ 3-fold above controls in samples (**Figures 3A, B**). In contrast, the Rac1•GTP levels in some patient samples were < 3-fold above and often below control levels (**Figures 3C, D**). We attribute the lower Rac1•GTP levels to several factors, including immunosuppression, an increased population of immature neutrophils (pandemic) in some patients, and pathogen-induced depletion of neutrophils (neutropenia) in others (Seigel et al., 2012; Liu et al., 2022). The observed ‘immunosuppression’ was not limited to the rarer bacteremia-inducing species (*P. aeruginosa*, *S. lugdunensis)* shown in **Figures 3C, D** but was also present in patients infected by the more common species causal in bacteremia (*E. coli* and *S. aureus*), where the Rac1•GTP levels across cases, span a wide range (**Figure 4**). These data suggest that Rac1•GTP levels act as a sensitive indicator of immune responsiveness for identifying different classes of bacteria.

The immune response to infection changes white blood cell counts and provides an additional dynamic and quantitative measure of immune system function in patients with bacteremia. Different bacterial strains may affect these cell counts in various ways. For instance, when neutrophils ingest microbes, such as *E. coli*, *P. aeruginosa*, and *S. aureus, they* trigger “phagocytosis-induced cell death”, resulting in apoptosis (Kobayashi et al., 2018). For classification purposes, neutrophil counts in infected patients were sorted into three categories relative to controls: **a**) above the normal range (neutrophilia), **b**) within the range of control counts, and **c**) below the range of control counts (neutropenia) (**Figure 4A** **).** Some cases were distributed across more than one category, e.g. *(E. coli* (**a**, **b**, **c**); *K. pneumoniae* (**b**, **c**); *S. pyogenes* (**a, b**) and *S. aureus* (**a**, **b**)). More detailed analyses of infections caused by gram-negative bacteria *(E. coli and K. pneumoniae)* and gram-positive bacteria *(S. aureus and S. pyogenes*) with the highest numbers of patients are shown in **Figure 4B**. The highest levels of Rac1•GTP are notably produced in category **c** patients, who also exhibited extremely low neutrophil counts due to infections caused by gram-negative bacteria such as *E. coli* and K. *pneumoniae.* In contrast, infections caused by gram-positive bacteria, such as *S. aureus*, were more likely to cause neutrophilia, though the neutrophils are inactive. Our analyses of the collective data indicate that different bacterial species have unique effects on innate and adaptive immune functions.

### Patterns of immune response variables depend on the species of bacteria

Our study is founded on the premise that the expression patterns of specific immune response variables—Rac1·GTP (in PMN or PBMC), neutrophils, lymphocytes, and monocytes—are unique to each species and sufficiently distinct to identify bacterial species.

**Figure 5:**
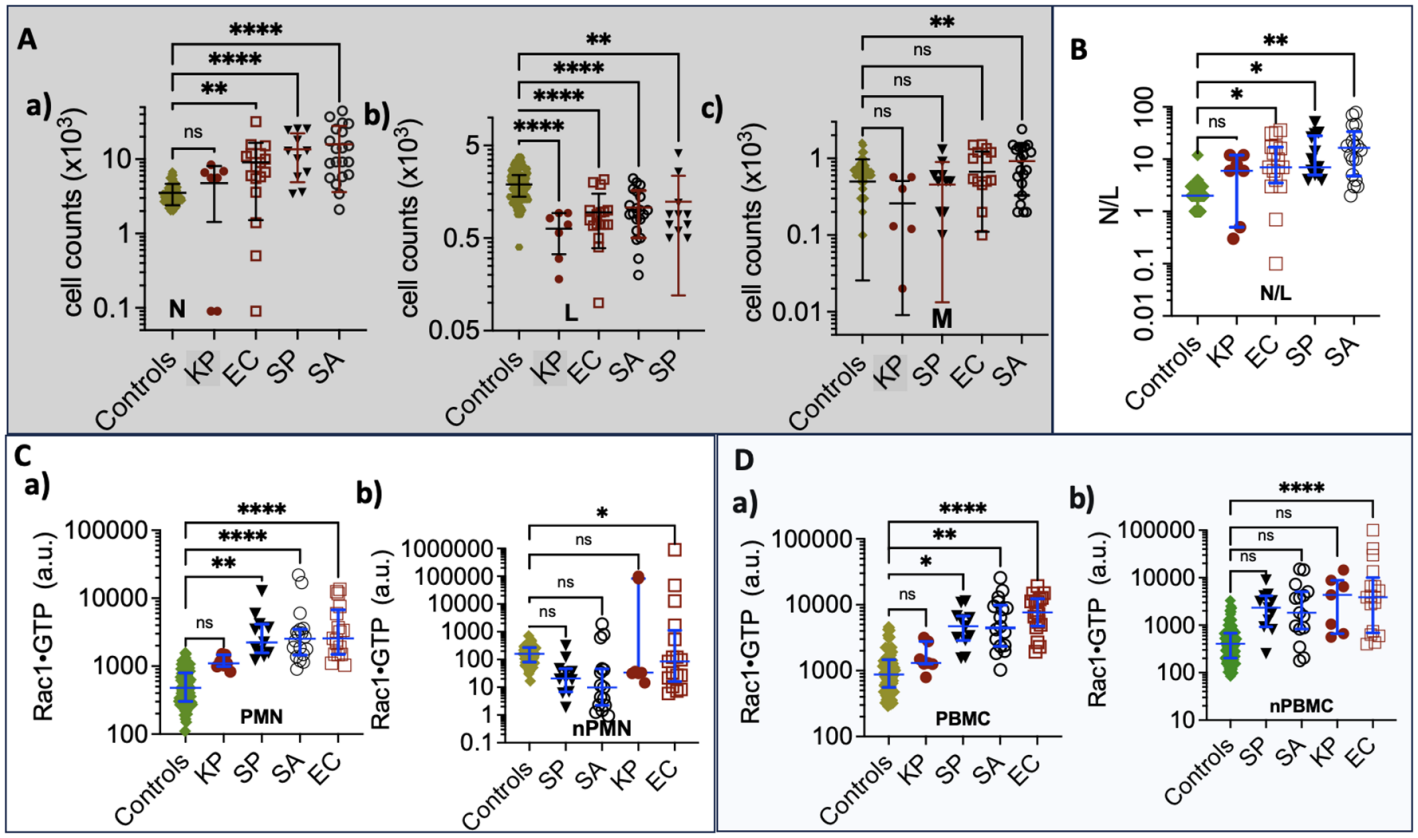
**(A)** Complete Blood Count graphs of **(a)** Neutrophil counts **(b)** Lymphocytes **(c)** Monocytes derived from samples collected from control and bacteremia cases due to *E*. *coli* (EC*)*, *K*. *pneumoniae*, (KP) *S. aureus* (SA), and *S. pyogenes* (SP) infections. **(B)** Plots of Neutrophil/Lymphocyte Ratio (N/R) in control and bacteremia cases. **(C)** Rac1•GTP is measured in neutrophil lysates (PMN); **(a)** aggregate GTPase activity of the total cell population. **(b)** GTPase activity per cell for each sample. **(D)** Rac1•GTP measured in PBMC lysates. **(a)** aggregate GTPase activity of total cell population. **(b)** GTPase activity per cell for each sample; * p ≤ 0.05; **p ≤ 0.01; *** p ≤ 0.001; ****p ≤ 0.0001. Statistics ordinary one-way ANOVA multiple comparisons (Dunnett); horizontal line indicates the median with interquartile range. ns: not significant.

*Neutrophils (N).* Unlike other pathogens, *K. pneumoniae* infection causes minimal changes in neutrophil counts relative to controls (see panel **a** in **Figure 5A**). *E. coli* and *K. pneumoniae* result in small numbers of neutropenic patients. The gram-positive *S. aureus* and *S. pyogenes* elicit significantly higher neutrophil counts than controls. Thus, *K. pneumoniae* is separated from the other species. *Lymphocytes (L).* Lower lymphocyte counts in all groups are attributed to their redistribution within the lymphatic system and the acceleration of apoptosis (Lowsby et al., 2015) (see panel **b** in **Figure 5A**). Consequently, lymphocyte counts are generally not discriminative among species.

*Monocytes (M).* Patients infected with *S. aureus* show significantly increased monocyte levels compared to others (see panel **c** in **Figure 5A**). Monocytes play a key role as an early defense mechanism against *S. aureus*, (Mellergaard et al., 2020) making them a valid discriminant factor for identifying *S. aureus* infections among *the* declared group of unknowns.

Neutrophil-to-Lymphocyte ratio (N/R)*.* In **Figure 5B**, we analyzed the balance between acute inflammation and adaptive immunity using the N/R. The N/R indicates systemic inflammation based on complete blood count values. (Buonacera et al., 2022) Changes in N/R can arise from an increase in neutrophils, a decrease in lymphocytes, or a combination of both. As the disease progresses, neutrophil counts tend to increase while lymphocyte counts decrease. The N/R value for *K. pneumoniae* is similar to that of the control group; in contrast, patients infected with *E. coli*, *S. pyogenes*, and *S. aureus* exhibit significantly higher N/R values than the controls. Our data show that the gram-positive bacteria *S. pyogenes* and *S. aureus* significantly elevate neutrophil counts, resulting in a higher N/R. However, *S. aureus* produces chemotaxis inhibitory proteins that impair neutrophil function, (Kobayashi et al., 2018) distinguishing *S. aureus* from *E. coli* and, to a lesser extent, *S. pyogenes* infections.

*Rac1•GTP.* In **Figures 5C, D**, the Rac1•GTP content in *K. pneumoniae* infections is comparable to the control group, while *E. coli* shows the highest levels among the tested groups. To assess the unit activity of the cell populations, we normalized Rac1•GTP content to the number of cells. Only the cells activated by *E. coli* exhibited a significant difference in Rac1•GTP content per unit cell compared to infection by the other bacterial species.

In summary, *K. pneumoniae* is distinguishable from its cohort due to its similarity to the controls, while *E. coli stand*s out because of significant Rac1•GTP expression in infected hosts. The gram-positive bacteria *S. aureus* and S*. pyogenes* can be differentiated from the gram-negative bacterium *E. coli* by their association with neutrophilia and low levels of Rac1•GTP, which result from impaired neutrophil function (Kobayashi et al., 2018). The link between *S. aureus and* elevated monocyte counts (Mellergaard et al., 2020) further distinguishes it from *S. pyogenes*.

### Classification and discrimination of cases based on bacterial species

**Figure 6:**
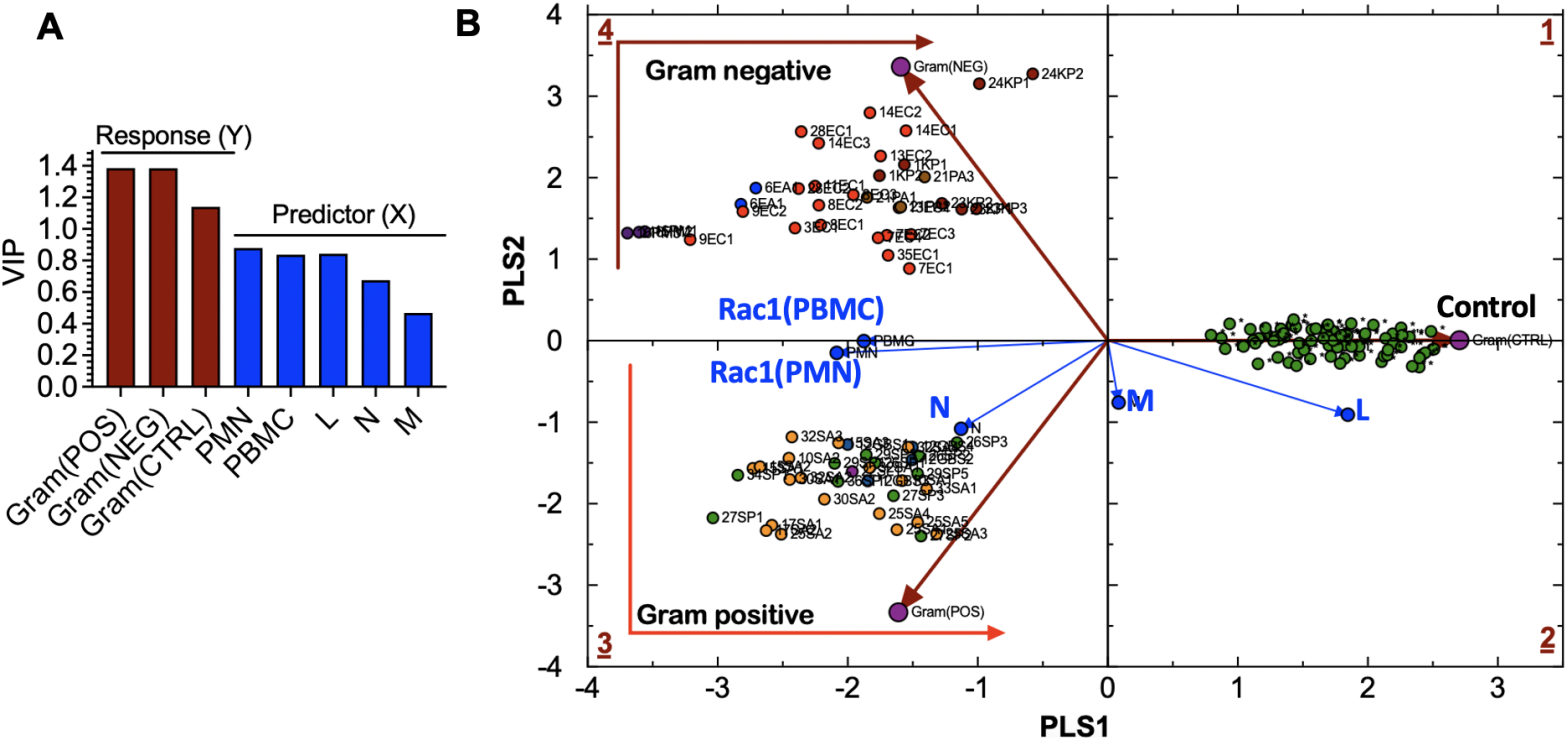
**(A)** Variable influence on projection (VIP) of: **(a)** bacteria-related response variables, including Gram(POS), Gram(NEG), control (CTRL) samples, and **(b)** experimental predictor variables, including peripheral blood mononuclear cells (PBMC), lymphocytes (L), monocytes (M), neutrophils (N)). **(B)** Partial Least Squares-Discriminant Analysis (PLS-DA) showing quadrant distribution of control samples and samples from patients with bacteremia caused by gram-negative (Gram(POS) and gram-positive (Gram(NEG) species. Response loadings (brown) are closely correlated with PC scores. Experimental loadings (blue) show a correlation of lymphocytes (L) and neutrophils (N) with control samples and gram-positive bacteria, respectively. *S. aureus* infections elicit the production of high neutrophil counts; thus, the correlation with N. Gram-negative species: *E*. *coli* (EC) are bright red; *E*. *aerogenes* (EA) are blue; *K*. *pneumoniae* (KP) are dark red; *P. multocida (*PM) are dark purple; *P. aeruginosa* (PA) are brown. Gram-positive species: *S. aureus* (SA) is orange, MRSA is light purple, and *S. pyogenes* (PA) is green. Controls: uninfected cases.


To support our qualitative model, we used multivariate statistical analysis to identify bacteria-specific immune response patterns, helping to differentiate and group patients with the same infection using Partial Least Squares Discriminant Analysis (PLS-DA). To conduct a PLS-DA analysis, we separated the bacteria-free controls and cases infected with either gram-positive or gram-negative bacteria into “tolerance volumes” (Eriksson et al., 1999). Using multiple linear regression based on Equation 1, we established spatial separation between uninfected cases and patients infected with gram-positive or gram-negative bacteria (**Figure 6A**) (Mendez et al., 2020).

(1) $$\begin{array}{c} {\text{s}}_{\text{ip}}={\text{ν}}_{(\text{RAC}1(\text{PBMC}),\text{p}}•{\left[\text{ RAC}1(\text{PBMC})\right]}_{\text{i}}+{\text{ν}}_{(\text{RAC}1(\text{PMN}),\text{p}}•{\left[\text{ RAC}1(\text{PMN})\right]}_{\text{i}}\\ +{\text{ν}}_{(\text{N})\text{p}}•{\left[\text{ N}\right]}_{\text{i}}+{\text{ν}}_{(\text{N})\text{p}}•{\left[\text{ L}\right]}_{\text{i}}+{\text{ν}}_{(\text{N})\text{p}}•{\left[\text{ M}\right]}_{\text{i}} \end{array}$$

The variable influence on projection (VIP) graph shows the relative weights of (X) variables used to predict the sample association with gram-positive and gram-negative species infections and control samples. X-variables with VIP scores, such as Rac1•GTP levels in PMN (Rac1(PMN)), Rac1•GTP in PBMC (Rac1(PBMC)), and L, are more significant in discriminating between classes in PLS-DA as shown for the response (Y) variables represented by gram bacteria class and controls (**Figure 6B**). The Rac1(PMN or PBMC) vectors bisect the classification clusters, indicating the equal influence of bacterial species. The strong correlation between the N and gram-positive species is consistent with the prevalence of high neutrophil counts associated with gram-positive bacterial infection. Neutrophils are the primary immune cells that respond to *S. pyogenes*, also known as gram-positive Group A streptococcus (Edwards et al., 2018).

The separation of bacteria by classification is linked to the signature immune response mechanisms of gram-positive and gram-negative species. TLRs recognize pathogen-associated molecular patterns on the surface of bacteria and are essential to innate immune defenses. Gram-positive bacteria cells have peptidoglycan cell walls detected by TLR2 (Koch and Zacharowski, 2009; Reynolds et al., 2012), while gram-negative bacteria cells have thin peptidoglycan and lipopolysaccharides in their outer membranes, activating TLR4 and sometimes TLR2/5. Pathogen recognition receptors regulate Rac1 GTPase-mediated leukocyte recruitment by activating class-specific signaling pathways that produce cytokines and other molecules (Jones et al., 1998; Ridley, 2001; 2006), thereby orchestrating the innate and adaptive immune response to infection.

To set the stage for predicting the identity of specific infectious agents ahead of or without positive blood culture confirmation, we sorted patient samples infected with *E. coli*, *K. pneumoniae*, *S. pyogenes*, and *S. aureus* into ‘separate tolerance’ volumes (Eriksson et al., 1999). We excluded samples of rare bacteria, of which only four or fewer were available per species, e.g., *P. multocida*, *P. aeruginosa*, and Group B streptococcus. However, Equation 1 in the PLS-DA model failed to resolve the intra-class bacterial species. We reasoned that our five original variables were not sufficiently robust to resolve intra-class species differences. We, therefore, increased our variables to eight by adding white blood cell count (WBC), the sum of lymphocytes and monocytes (L+M), and the N/L (Equation 2), which represents the balance between acute inflammation and adaptive immunity (Seigel et al., 2012).


(2) $$\begin{array}{c} {\text{s}}_{\text{ip}}={\text{ν}}_{(\text{RAC}1(\text{PBMC}),\text{p}}•{\left[\text{ RAC}1(\text{PBMC})\right]}_{\text{i}}+{\text{ν}}_{(\text{RAC}1(\text{PMN}),\text{p}}•{\left[\text{ RAC}1(\text{PMN})\right]}_{\text{i}}+{\text{ν}}_{(\text{N})\text{p}}•{\left[\text{ N}\right]}_{\text{i}}\\ +{\text{ν}}_{(\text{L})\text{p}}•{\left[\text{ L}\right]}_{\text{i}}+{\text{ν}}_{(\text{M})\text{p}}•{\left[\text{ M}\right]}_{\text{i}}+{\text{ν}}_{(\text{WBC})\text{p}}•{\left[\text{ WBC}\right]}_{\text{i}}+{\text{ν}}_{(\text{L}+\text{M})\text{p}}•{\left[\text{ L}+\text{M}\right]}_{\text{i}}+{\text{ν}}_{(\text{N}/\text{L})\text{p}}•{\left[\text{ N}/\text{L}\right]}_{\text{i}} \end{array}$$

**Figure 7:**
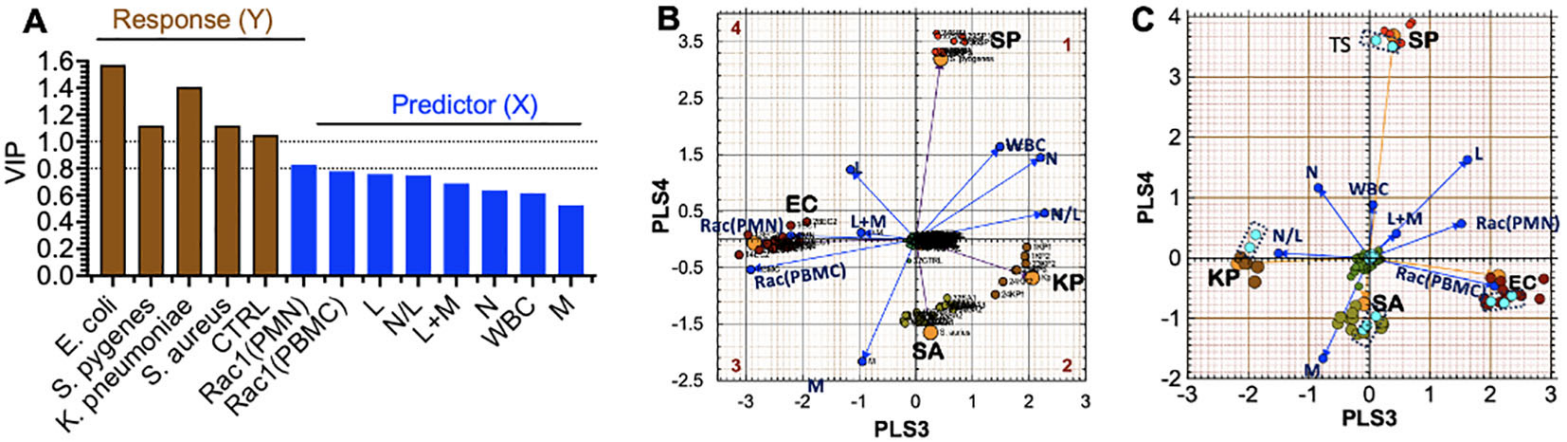
Partial Least Squares-Discriminant Analysis (PLS-DA) shows a quadrant distribution of controls and patients based on bacterial species found in blood culture. **(A)** Variable influence on projection (VIP) of response variables (bacteria-species) and experimental variables (PMN, PBMC, lymphocytes (L), monocytes (M), neutrophils (N)). The relative influence of loadings is derived from 8 variables in Equation 1. The increase in weight differential in the response variables (Y) yields greater separation. **(B)** The Case ID letters refer to infecting pathogen SA: *S. aureus*, SP: *S. pyogenes*, EC: *E*. *coli*, KP*: K. pneumoniae*. Controls (center of the graph) Observed (principal components) and predicted (bacteria loadings) plots for each response show good agreement. **(C)** Cross-validation to confirm the PLS-DA classification model using the training set and test set (teal data points inside dotted rectangles) in predicting class memberships with the test set samples; Q^2^ for the new model is 0.88, where *Q* ^2^ evaluates the error between the predicted response variable y and the known y.

Adding extra experimental variables enhanced the (VIP) scores (**Figure 7A**), resulting in improved separation of the intra-class bacterial species on the discriminant hyperplane (PLS3 vs. PLS4) (**Figure 7B**). The L+M loadings vector was correlated with *E. coli* infections. In contrast, the N/L and WBC loadings were correlated with *K. pneumoniae.* Gram-negative bacteria (*E. coli and K. pneumoniae*) were distributed along the PLS3 component axis, whereas the gram-positive bacteria (*S. aureus* and *S. pyogenes*) were associated with the PLS4 axis. Rac1(PMN) and Rac1(PBMC) had the most significant impact on identifying *E. coli* as the infectious agent, which is consistent with the tendency of *E. coli* bacteria to elicit more robust innate and adaptive immune responses than the other bacterial species in this study (*cf*. **Figures 5C, D**).

External Set Validation. For PLS-model predictions, a new observation is considered to be similar to the training set if it is located inside the *tolerance volume*. To achieve this goal, we randomly selected 2-3 samples from each group infected by a distinct pathogen and de-identified the species. Instead, we used the PLS-DA model to predict the unknown species based on the relative data fit to the existing *tolerance* volumes. The test set was only used to measure the performance of the model. The prediction error measure Q^2^ is the default parameter used in PLS-DA discriminations, which focuses on how well the class label can be predicted from test samples and new data. Q^2^ depends on the inter-class separation and the intra-class variability. The validation model showed that the EC and *K. pneumoniae* coordinates were rotated by 180° along the PLS3 axis compared to the original model (as indicated in **Figure 7C**). Despite this change, our model could correctly classify all test samples, which demonstrated high accuracy with a predictive ability of Q^2^ = 0.88.

## Discussion

Our study aimed to determine if multivariate analysis of immune variables based on complete white blood cell counts and Rac1•GTP levels in innate and adaptive immune cells—are adequate to identify the bacterial species associated with bacteremia in various patients. Rac1 plays several crucial roles related to host immunity by: a) mobilizing leukocytes, thereby triggering the expression of genes that code for proinflammatory cytokines, chemokines, cell adhesion molecules, and immunoreceptors, b) activating transcription factors that promote cell growth and expansion of antigen-specific lymphocytes (Turner et al., 1998), and c) regulating immune cell motility, adhesion, and transcription. Thus, Rac1 integrates upstream and downstream GTPase responses that lead to immune cell motility-driven cytoskeletal dynamics, adhesion, and transcription (Jones et al., 1998; Ridley, 2000). Elevated or suppressed levels of Rac1•GTP, along with a complete blood cell count, can help identify the species causing bacteremia. As an immune signaling node, Rac1 GTPase activity measurement avoids the need and costs of measuring multiple variables in real-time (*cf.* **Figure 7**) and subsequent analysis on various platforms at high costs. The differential diagnosis of an infection involves determining which pathogen is most likely causing the disease. Bacterial strains evade host innate immune responses by blocking chemo-attractants and inhibiting neutrophil mobilization (Surewaard et al., 2013; de Oliveira et al., 2016) (Haas et al., 2004), often targeting GTPase-dependent immune mechanisms (Mao and Finnemann, 2015). This suppression can weaken the adaptive immune response (Kobayashi et al., 2018). Our study highlights the critical role of Rac1•GTP and associated white blood cell counts in segregating patient samples into clusters based on unique immune response patterns associated with different bacterial species.

Our discussion is limited to the most abundant species in our study cohort: *E. coli*, *S. aureus*, *S. pyogenes, and K. pneumoniae*. Patients with an *E. coli* infection typically exhibit high neutrophil counts and Rac1•GTP. In contrast, those with an *S. aureus* infection have low Rac1•GTP levels, even though they may show high neutrophil counts. This is because *S. aureus* impairs neutrophil function and produces anti-inflammatory metabolites (Frodermann et al., 2011). Depleted cell counts in late-stage samples occur due to cell-ingested EC (1, 2, 3) and KP (1, 2) (in **Figure 4B**), initiating “phagocytosis-induced cell death” to suppress an overactive immune system. *S. aureus* is a significant cause of bacteremia and sepsis, leading to serious infections, including central line-associated bloodstream infections, skin infections, pneumonia, and toxic shock syndrome (Haslinger-Loffler et al., 2005; Greenlee-Wacker et al., 2014). Therefore, the ability to discriminate *E. coli* and *S. aureus* infections is a significant advance.

*S. pyogenes* is the leading cause of maternal sepsis (puerperal sepsis) occurring within six weeks of childbirth, significantly contributing to maternal and infant mortality worldwide (Grondahl-Yli-Hannuksela et al., 2021). Invasive *S. pyogenes* can lead to Toxic Shock Syndrome, which occurs in 20% to 33% of pregnancy-related cases. This condition is responsible for approximately 50% of fatal cases, sometimes within just 24 hours of the onset of symptoms. Therefore, prompt diagnosis and the early administration of appropriate antibiotic therapy are essential (Hamilton et al., 2013; Stevens and Bryant, 2023). Elsewhere, pathogen-specific bacteremia urinary tract infections (Al-Hasan et al., 2010) account for 1-6% of healthcare visits, where the treatment cost in the US alone is approximately $1.6 billion annually (Sulham and Hammelman, 2021). Globally, 404.61 million urinary tract infections were reported in 2019, with *E. coli and K. pneumoniae* as the most common cause (Yang et al., 2022). *K. pneumoniae* can also cause severe infections in newborns, cancer patients, and those with weakened immune systems (Follador et al., 2016; Chang et al., 2021). Treating *E. coli and K. pneumoniae* bacteremia can be challenging because of the overuse of antibiotics targeting *E. coli*, which is the more prevalent pathogen (Girometti et al., 2014; Xu et al., 2015; Sung et al., 2021). This makes it crucial to identify the pathogen accurately and rapidly to prevent prescribing unnecessary antibiotics and limit antibiotic resistance. As shown in **Figure 7**, the measure of Rac1•GTP using the G-Trap assay can accurately differentiate *S. aureus*, *S. pyogenes, E. coli*, and *K. pneumoniae* with distant separations. This allows healthcare workers to customize treatment and adjust the antibiotic regimen in real time based on the patient’s immune response.

We acknowledge that the limitations of our study will be addressed in future research to verify the effectiveness of the machine-learning approach in managing antimicrobial usage. Our data set is small. However, in limited numbers for the rare species in our cohort, we could still accurately identify the class (gram-positive or gram-negative) of bacteria species. We achieved our primary objective of developing a proof-of-concept assay to evaluate patient immune functionality as a biomarker for the rapid assessment of presumptive bacteremia. Since this was a masked pilot study, characterizing the trajectory from bacteremia to recovery was not possible, and it presented limited individualized insight into intervention strategies due to the minimal dataset and comprehensive access to clinical patient variables.

In sum, our study validated and demonstrated that quantitative assessment of immune cell activation through white blood cell counts and Rac1•GTP levels is associated with the convergence of leukocyte activation signaling pathways (Infante and Ridley, 2013). G-Trap tests can help narrow antibiotic prescriptions based on local antibiograms and streamline differential diagnosis (Southwick, 2020a; b) The correlation between infection of named bacterial species and infected patients can inform antibiotic prescription and de-escalation in real-time.

## Materials and methods

### G-Trap effector beads

G-Trap is a flow cytometry assay that uses bead-based protein immobilization to quickly measure the activity status of GTPases in cells or tissue lysates. (Simons et al., 2018) Multiplex format assay bead sets are functionalized with different glutathione-S-transferase-effector proteins at the surface of each set. Users can incubate bead sets individually or in a multiplex format with lysates for rapid, selective capture of active, GTP-bound GTPases from a single sample. Flow cytometry detects the bead-borne GTPase and quantitatively measures the amount of active GTPase per bead using labeled secondary antibodies.

### Effector proteins

We used the glutathione-S-transferase-effector protein chimera p21-activated kinase protein binding domain, a Rac1 effector protein purchased from Millipore Sigma.

### Antibodies

Monoclonal Rac1 antibodies (Cat. # ARC03) were purchased from Cytoskeleton.

### Buffers

*The* 2X Radioimmunoprecipitation Assay Buffer, commonly known as *RIPA, consists of* 100 mM Tris (tris(hydroxymethyl)aminomethane) titrated with HCl to pH 7.4, 300 mM NaCl, 2 mM ethylenediaminetetraacetic acid, 2 mM NaF, 2 mM Na_3_VO_4_, 2% NP-40, and 0.5% sodium deoxycholate. Just before adding it to the culture medium, include 2 mM phenylmethylsulfonyl fluoride and 2X protease inhibitors. *HHB Buffer contains* 7.98 g/L HEPES (2-[4-(2-hydroxyethyl)piperazin-1-yl]ethanesulfonic acid, Na salt), 6.43 g/L NaCl, 0.75 g/L KCl, 0.095 g/L MgCl_2,_ and 1.802 g/L glucose. The *HPSMT buffer*, an intracellular mimic, is made up of 30 mM HEPES, pH 7.4, 140 mM KCl, 12 mM NaCl, 0.8 mM MgCl_2_, and 0.01% Tween 20 (Polyethylene glycol sorbitan monolaurate).

### Study design

The University of New Mexico Health Sciences Center Human Research Protections Office Institutional Review Board approved this study (UNM IRB#18-068). The study was classified as category (5), which refers to data, documents, records, and specimens (blood samples, in our case). It is important to note that our Institutional Review Board approval for the pilot study did not include access to comprehensive clinical data. After a positive diagnosis of a non-infectious disease state or confirmed bacteremia, we collected residual blood samples directly from the clinical lab. We obtained blood samples from 120 patients with non-infectious diseases. When patients showed signs of bacteremia, their physicians ordered daily blood samples to test for bacterial growth. Any extra samples were stored in the clinical laboratory at 4°C until a bacterial culture was confirmed. In this manner, serial blood test samples (61 samples) from 28 patients with a confirmed diagnosis of bloodstream infection were collected after standard-of-care testing at TriCore Reference Laboratories. TriCore Reference Laboratories provided the investigators with complete blood counts with differential for each subject.

### Isolation of PBMCs and PMNs diagnostic samples

Whole blood samples were kept at 4°C. PBMCs consist of lymphocytes (T, B, and natural killer (NK) cells) and monocytes. PMNs, comprising primarily neutrophils with a small fraction of eosinophils, basophils, and mast cells, were isolated using a Ficoll-based density gradient using 1 ml of blood for all samples. The PBMC and PMNs layers were carefully recovered in 800 µl volumes, mixed with 800 µl PBS, centrifuged, and removed the excess solution, leaving the pellet in 25 µl volume. The pellet was lysed, and soluble Rac1-GTP was recovered using PAK1-functionalized beads, then labeled with anti-Rac1 primary and fluorescent secondary antibodies before being analyzed using a flow cytometer.

### Functional assessment of PBMCs and PMNs with Rac1•GTP binding assays

The pelleted cells are lysed by adding 25 µl 2X Radioimmunoprecipitation assay buffer at 4°C and centrifuged at 4°C in a cold room. Forty-five microliters of cleared lysate were analyzed for Rac1•GTP content using G-Trap beads functionalized with PAK1 effector beads, (Simons et al., 2018) utilizing an Accuri C6 flow cytometer equipped with BD Accuri C6 Software for data collection and analysis. We used the CBC with differential data from Tri-Core to determine how much Rac1•GTP was produced by single cells.

### Multivariate data analysis: model training, validation, and testing

Due to lack of complete blood count (CBC) data in 6 cases, the final data set used for the *multivariate analysis*, drawn from 22 patients, consisted of 61 data points: (*K. pneumoniae* (7)) *S. aureus* (16), MRSA *(2), S. pyogenes* (10)*, E. coli (16), P. aeruginosa* (3), *P. multocida (3)*, Group B streptococcus (4), *E. aerogenes* (2)). For control experiments, 80 samples from 80 patients were treated for non-infectious disease causes.

We used GraphPad Prism 10.0 (GraphPad Software, La Jolla, CA) for principal component analysis and SIMCA-P 9.0 software (Eriksson et al., 1999) (Umetrics, Umeå, Sweden) for partial least squares discriminant analysis (PLS-DA). PLS-DA is a classification method similar to a supervised application of principal component analysis. In PLS-DA, one knows the class membership (bacterial species) contained in the algorithm’s categorical variables. The purpose of PLS-DA is to predict sample class membership in matrix **Y** based on measured immune response data in matrix **X**. PLS-DA achieves this by reducing the dimensionality of the data through a transformation that results in latent variables (LVs). These LVs are derived from linear combinations of the original immune variables (N, L, M Rac1(PMN, Rac1(PBMC))) that attempt to explain the maximum covariance between **X** and **Y**. In other words, PLS-DA uses analytes with significant quantitative variations and tries to correlate them to the sample class information in **Y**. Other meaningful results are scores and loadings that describe the samples and the immune variables, respectively. A scores plot ideally shows sufficient separation of the class input as a part of the analysis (i.e., the responses in **Y**).

On the other hand, a loadings plot would demonstrate the variables (i.e., N, L, M Rac1(PMN, Rac1(PBMC))) that significantly differentiate the sample classes (i.e., the spatial distribution of bacterial species). Samples have scores on each determined LV, and the immune variables have loadings for each LV. These scores and loadings are used to estimate **Y**. Therefore, PLS-DA helps predict the sample membership for both calibration/training sets of samples used to build the model and new samples in the future, where knowledge of class membership is unknown.

#### Validation and testing

The Q^2^ is a prediction error measure used in PLS-DA discriminations to evaluate how well the class label can be predicted from new data. This study used a limited data set of test samples (10 controls and 2-3 samples chosen from each bacteremia species). The Q^2^ diagnostic statistic validates PLS-DA models. This is done by evaluating the error between the predicted categorical variable (ŷ) and the known variable (y). Q^2^ is defined as 1 minus the ratio of the Prediction Error Sum of Squares (PRESS) over the Total Sum of Squares (TSS) of the response vector y (Q^2^ = 1 - PRESS/TSS) (Cruciani et al., 1992).

## Data Availability

The raw data supporting the conclusions of this article will be made available by the authors, without undue reservation.
